# The Challenges of Colorectal Cancer Surgery during the COVID-19 Pandemic in Romania: A Three-Year Retrospective Study

**DOI:** 10.3390/ijerph192114320

**Published:** 2022-11-02

**Authors:** Cristi Tarta, Marco Marian, Marco Capitanio, Flaviu Ionut Faur, Ciprian Duta, Razvan Diaconescu, Anca Monica Oprescu-Macovei, Bogdan Totolici, Amadeus Dobrescu

**Affiliations:** 1 Department X, 2nd Surgical Clinic of General Surgery, “Victor Babes” University of Medicine and Pharmacy, 300041 Timisoara, Romania; 2 Department of Gastroenterology, “Victor Babes” University of Medicine and Pharmacy, 300041 Timisoara, Romania; 3 Department of General Surgery, Faculty of Medicine, “Vasile Goldis” Western University of Arad, 310025 Arad, Romania; 4 Department of Gastroenterology, Emergency Hospital “Prof. Dr. Agripa Ionescu”, University of Medicine and Pharmacy “Carol Davila”, 050474 Bucharest, Romania

**Keywords:** SARS-CoV-2, COVID-19 pandemic, colorectal cancer, cancer epidemiology, general surgery

## Abstract

The predictions on the influence of the SARS-CoV-2 pandemic on access to medical services in Romania predicted a 35% drop in oncological hospitalizations in 2020 compared to the previous decade, raising the hypothesis that patients with colorectal cancer can become indirect victims of the ongoing pandemic. Therefore, the aim of the current research was to observe how the COVID-19 pandemic influenced colorectal cancer surgery in Romania, to determine the level of addressability towards specialized care, to compare the cancer staging between the pandemic and pre-pandemic periods, and to observe the risk factors for disease progression. This retrospective study was spread over three years, respectively, from March 2019 to March 2022, and included a total of 198 patients with a history of colorectal cancer surgery. It was decided to perform a parallel comparison of 2019, 2020, and 2021 to observe any significant changes during the pandemic. Our clinic encountered a significant decrease in all interventions during the pandemic; although the number of CRC surgeries remained constant, the cases were more difficult, with significantly more patients presenting in emergency situations, from 31.3% in 2019 to 50.0% in 2020 and 57.1% in 2021. Thus, the number of elective surgeries decreased significantly. The proportion of TNM (tumor-node-metastasis) staging was, however, statistically significant between the pre-pandemic and pandemic period. In 2019, 13.3% of patients had stage IIa, compared with 28.8% in 2020 and 13.1% in 2021. Similarly, the proportion of very advanced colorectal cancer was higher during the pandemic period of 2020 and 2021 (12.0% in 2019 vs. 12.5% in 2020 and 25.0% in 2021), which was represented by a significantly higher proportion of patients with bowel perforation. Patients with an advanced TNM stage had a 6.28-fold increased risk of disease progression, followed by lymphovascular invasion (HR = 5.19). However, the COVID-19 pandemic, represented by admission years 2020 and 2021, did not pose a significant risk for disease progression and mortality. In-hospital mortality during the pandemic also did not change significantly. After the pandemic restrictions have been lifted, it would be advisable to conduct a widespread colorectal cancer screening campaign in order to identify any instances of the disease that went undetected during the SARS-CoV-2 pandemic.

## 1. Introduction

Colorectal cancer is still the second largest cause of mortality from cancer in the United States of America and the third most common cause in the European Union [1,2]. According to the most recent recommendations, patients are checked on a regular basis using either endoscopic procedures such as flexible sigmoidoscopy and colonoscopy, or stool-based testing [3]. Similarly to the European guidelines, screening for colorectal cancer in the US is advised in people aged 50 to 75 years old, as well as in persons aged 45 to 49 years old in particular cases with known personal and familial risks [4]. In addition, the US Preventive Services Task Force suggests that doctors selectively give colorectal cancer screening to persons between the ages of 76 and 85 years old [5]. 

The SARS-CoV-2 pandemic had an important negative and severe influence on the operation of healthcare systems across the world [6]. As a result, several nations committed all of their available resources to the fight against the illness, with varying degrees of success. In order to contain the disease’s rapid spread across the country of Romania, the authorities put severe restrictions on public and social life. Patients and medical staff were prioritized above everything else throughout the process of putting in place stringent safety measures. In order to limit patients’ access to elective therapies and be ready for the arrival of COVID-19 patients, hospitals were reorganized [7]. As a direct consequence of this, the majority of hospitals were forced to reallocate their resources (equipment and workforce) to the treatment of COVID-19 patients, and many elective visits and surgeries that were already booked were pushed back for several weeks [8]. Because of their concerns about contracting an infection, a significant number of cancer patients felt hesitant to seek care, which put them in a position where their disease was more likely to develop. 

Similarly, several researchers exemplify how the burden of the COVID-19 pandemic created a negative syndemic situation for other groups of patients, such as patients with cancer [9,10]. Clustering of health disorders that are exacerbated by socioecological variables resulted in poorer outcomes among vulnerable groups. COVID-19’s biological interactions with existing communicable and noncommunicable disorders increases susceptibility to adverse health effects, while the biological interactions of the virus with social determinants of health and political variables have exhibited a similar pattern. These characteristics generate a range of potential consequences, such as an acceleration of acute situations for patient interventions.

In addition, because of the widespread dissemination of the new virus, surgical procedures have been significantly reduced since the majority of the hospital’s resources have been directed toward the isolation and treatment of COVID-19 patients [11]. Because of this, oncological management received more diminished attention than it should have. It was previously observed that a lot of cancer patients had delays in access to specialized care, many necessitating a different form of therapy than the one that was indicated initially [12]. In addition, it is believed that around 38% of all cancer surgical operations were canceled throughout the globe in the first 12 weeks of the epidemic [13]. This number comes from estimates made by several sources, with the earliest findings suggesting a considerable decrease in the incidence of colorectal cancer diagnosis during the early SARS-CoV-2 pandemic waves, delaying the treatment of these patients [14].

According to research on the influence of the SARS-CoV-2 pandemic on delivering medical care in Romania, there was a 35% drop in the number of oncological hospitalizations in the year 2020 when compared with the number of hospitalizations in the years before [15]. Since proper oncological care requires timely diagnosis and management, it was feared that oncological patients will become “silent collateral victims” of the pandemic due to observed delays [16]. Oncological diseases are notoriously difficult to diagnose in their early stages. After the first pandemic lockdown, national healthcare systems made gaining access to suitable treatment facilities for oncological patients a priority. However, under the standard of care that is currently being followed, elective procedures for intestinal malignancies are often postponed because there are not enough beds available in critical care facilities. There are over one million new instances of colorectal cancer diagnosed each year, making it the third most common form of cancer globally and one of the leading causes of mortality due to the disease [17].

This project aimed to give factual data on Romanian colorectal cancer patients during the SARS-CoV-2 crisis. The major aim was comparing the pre-pandemic and pandemic periods, detailing patients’ clinical characteristics, colon cancer diagnosis and progression, and available therapy. The secondary goal was in analyzing the result of patients treated at our center and the associated risk factors for disease progression during the pandemic. 

## 2. Materials and Methods

### 2.1. Research Design and Ethical Considerations

Patients were enrolled in the current retrospective cohort study if their hospital admission occurred between March 2019 and March 2022. The research was carried out at the University Clinic of General Surgery affiliated with the “Victor Babes” University of Medicine and Pharmacy in Timisoara. The administrative database that was used belonged to the clinic’s inpatient population and comprised both the study population and the features that were considered relevant. The major complaints, demographic information, surgeries performed, and other clinical data were identified from digital and paper records. These types of patient data were protected by privacy legislation and the patients’ agreement that were examined by certified physicians and other approved healthcare workers who were taking part in the present research project.

The general surgery clinic, as an auxiliary of Timis County Emergency Clinical Hospital “Pius Brinzeu,” operates under the laws of the Local Commission of Ethics that approves Scientific Research that functions based on the following regulations: (1) Article 167 of Law No. 95/2006, Art. 28, Chapter VIII of Order 904/2006; (2) the EU GCP Directives 2005/28/EC; (3) the International Conference on Harmonisation of Technical Requirements for Registration of Pharmaceuticals for Human Use (ICH). On 6 June 2022, the inquiry that is now underway was granted permission to proceed and was assigned the number 304.

### 2.2. Inclusion Criteria and Definitions

Patients identified with colorectal cancer surgery in their personal records were considered eligible for inclusion in the current study. They were identified by the International Classification of Diseases (ICD-10) diagnosis codes [18]. The objective of this study was to compare the cases of colorectal cancer from the pre-pandemic period with the COVID-19 pandemic period. The pre-pandemic was defined by the 12 months from March 2019 to March 2020, when the pandemic was officially declared in Romania [19]. The first year of the COVID-19 pandemic was from March 2020 to March 2021, while the second year comprised the following 12 months. The worldwide COVID-19 vaccination campaign was at its peak during the second year of the pandemic, allowing for governmental restrictions to be relaxed [20,21].

As a tertiary institution engaged in the treatment of colorectal cancer, all patients were referred to the institution under investigation by primary or secondary care providers. All subsequent eligible hospitalizations and follow-up investigations performed at the General Surgery Department were included in the current research. Other inclusion criteria were the patient’s age being at least 18 years old and the patient’s consent to engage in clinical research with complete private records. Patients were excluded if their medical records lacked vital information or the agreement documents. Patients without verifiable tests, diagnoses, or consent to participate in the study were excluded. Furthermore, patient data that lacked colorectal cancer staging was not included in the analysis.

Staging colorectal cancer is critical for identifying the neoplastic stage, assessing the degree of tumor cell infiltration, and establishing a precise patient profile. Considering tumor-node-metastasis (TNM) cancer staging that is used by both the American Cancer Society and the European Society of Coloproctology, colorectal cancer patients may be classified into several stages, ranging from stage 0 (intramucosal) to stage IV (metastatic disease) [22,23]. The diagnosis of colorectal cancer was considered only if a biopsy with a pathology result confirmed the malignancy, allowing the optimal treatment choices, prognosis, and survival rate to be determined. Tumor grading was performed under histopathology analysis, being graded from I to IV, as follows: Grade I—well differentiated, Grade II—Moderately differentiated, Grade III—Poorly differentiated; Grade IV—Undifferentiated or Anaplastic, according to the World Health Organization (WHO) criteria [24].

### 2.3. Variables

Members of the clinical teams gathered anonymized information on all colorectal cancer patients diagnosed throughout the research period. The following variables were collected: (1) background characteristics—age, age range, gender, body mass index, substance use behavior, place of origin, civil status, referral source, COVID-19 status; (2) colorectal cancer characteristics during the study period—number of comorbidities, anatomical distribution, tumor grading, TNM staging, tumoral aggressiveness; (3) colorectal cancer surgery features and outcomes—the proportion of CRCs, number of electives and emergency presentations, type of surgery performed, and outcomes; (4) risk factor analysis.

### 2.4. Statistical Analysis

IBM SPSS version 27.0 (SPSS Inc., Chicago, IL, USA) and Microsoft Excel (Microsoft Corp., Redmond, WA, USA). The representation of categorical variables was accomplished by absolute values and the frequencies of those values. A statistical examination of the proportions was carried out with chi^2^ and Fisher’s exact tests. A Shapiro–Wilk test was performed to determine the Gaussian distribution of data, and the ANOVA test was carried out to compare the means of Gaussian variables. Non-parametric variables were compared using the Kruskal–Wallis test. The Cox regression analysis was used to measure the effect of certain variables on disease progress, being adjusted for confounding factors. A level of significance of 0.05 was chosen as the threshold for the alpha value.

## 3. Results

**Figure 1:**
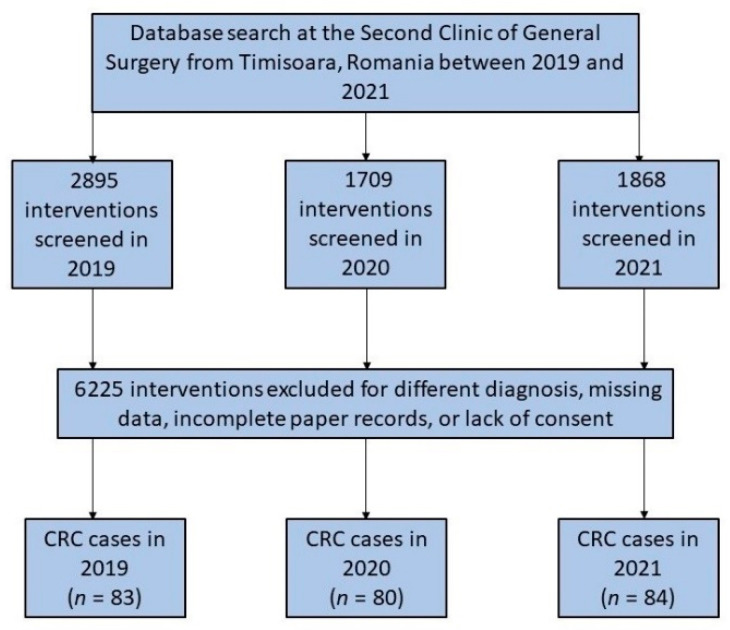
Diagram illustrating the inclusion of patients who had colorectal surgery over the 3-year research duration.

The number of all surgical procedures in the department had considerably fallen since March 2020, when the SARS-CoV-2 pandemic commenced in Romania. This was followed by the introduction of lockdown measures to prevent the spread of COVID-19. However, the number of new colorectal cancer interventions did not decrease and remained the same as the year before the start of the COVID-19 pandemic. During the pandemic, fewer new patients were reported or examined in outpatient settings, and more cases were emergency interventions (Figure 1). 

### 3.1. Patients’ Baseline Characteristics

Throughout the research duration of 36 months, a total of 247 patients were included in the study. As presented in Table 1, the average age of patients undergoing colorectal surgery was approximately 65 years, without significant differences before and during the pandemic. The majority of patients were men (62.7% in 2019 vs. 63.8% in 2020 and 51.2% in 2021) who were at the age of retirement. Generally, the patients were overweight, and the body mass index was higher than 25 on average between the three studied years, although without significant differences. Although the referral source did not differ significantly among the 36 months that were analyzed (*p*-value = 0.114), it was observed that there was a significant difference between groups. There was a statistically significant decrease in the number of referrals from primary care during the first year of the pandemic (2020), from 47.0% in 2019 to 31.3% in 2020 (*p*-value = 0.039). Only four patients had SARS-CoV-2 infection at the time of surgical intervention (two patients in 2020 and another two patients in 2021).

### 3.2. Comparison of Clinical and Oncological Characteristics

**Figure 2:**
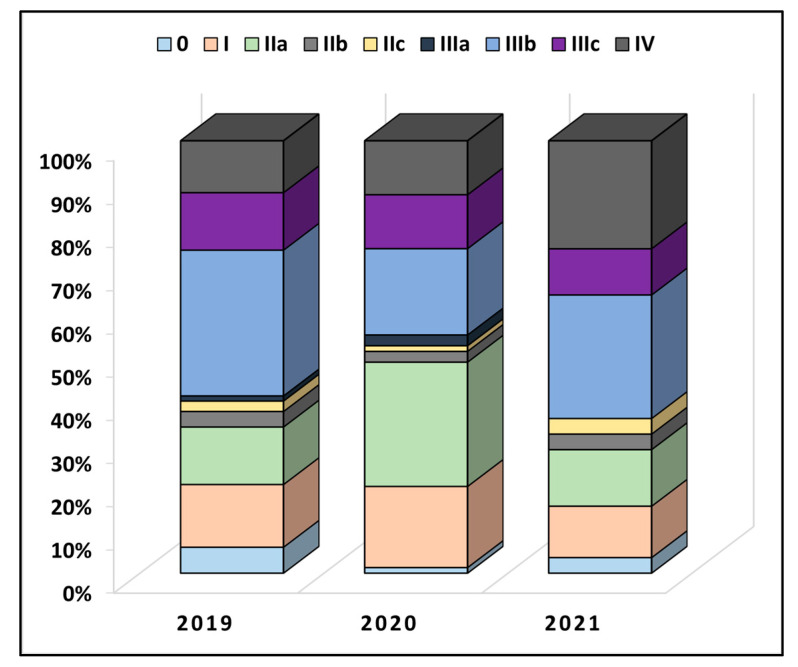
Comparison in TNM staging of colorectal cancer between patients undergoing surgical intervention before (2019) and during the COVID-19 pandemic (2020–2021).

Table 2 describes colorectal cancer characteristics between the years of study. It was observed that a majority of patients had at least two comorbidities at the time of admission (50.6% in 2019 vs. 55.0% in 2020 and 57.1% in 2021), without significant differences. The anatomical distribution of cancer had a colic location in 50 patients in 2019, 48 patients in 2020, and 44 patients in 2021 (*p*-value = 0.506). Less than 10% of all patients underwent radiotherapy or chemotherapy before the surgical intervention. The overall proportion of TNM staging was also not statistically significant between the pre-pandemic and pandemic period (*p*-value = 0.120), as seen in Figure 2. However, in 2019, 13.3% of patients had stage IIa, compared with 28.8% in 2020 and 13.1% in 2021 (*p*-value = 0.012). Similarly, the proportion of very advanced colorectal cancer was higher during the pandemic period of 2020 and 2021 (12.0% in 2019, vs. 12.5% in 2020, and 25.0% in 2021, *p*-value = 0.038). The lymphovascular invasion was also observed in more than 50% of the entire cohort, although without statistically significant differences.

### 3.3. Comparison of Outcomes and Interventions

A comparison of colorectal cancer surgery during the study period is presented in Table 3. Our clinic encountered a significant decrease in all interventions during the pandemic, although the number of CRC surgeries remained constant, thus significantly increasing the proportion of CRC interventions (2.86% in 2019 vs. 4.68% in 2020 and 4.49 in 2021, *p*-value = 0.001). Another important finding was that elective surgeries decreased significantly during the COVID-19 pandemic, from 68.7% in 2019, to 50.0% in 2020 and 42.9% in 2021 (*p*-value = 0.012), respectively. The proportion of patients with emergency presentation was also significantly higher during the pandemic, and the number of perforated bowels increased dramatically from 0 in 2019 to 4 in 2020 and 6 in 2021. The number of laparoscopic interventions also decreased during the pandemic, likely linked to the increase in emergent cases that lack proper preparedness before the intervention and contraindication of the laparoscopic approach. It was observed that a higher proportion of colostomy procedures were required during 2020 and 2021 compared to the pre-pandemic year 2019, increasing from 13 (15.7%) to 22 and 23 (27.3%, 27.4%), respectively. However, the between-groups comparison resulted in a non-significant *p*-value. Although the proportion of emergency situations increased during the pandemic, the in-hospital mortality rate did not differ significantly, similar to the disease progression at six months.

### 3.4. Risk Factor Analysis

**Figure 3:**
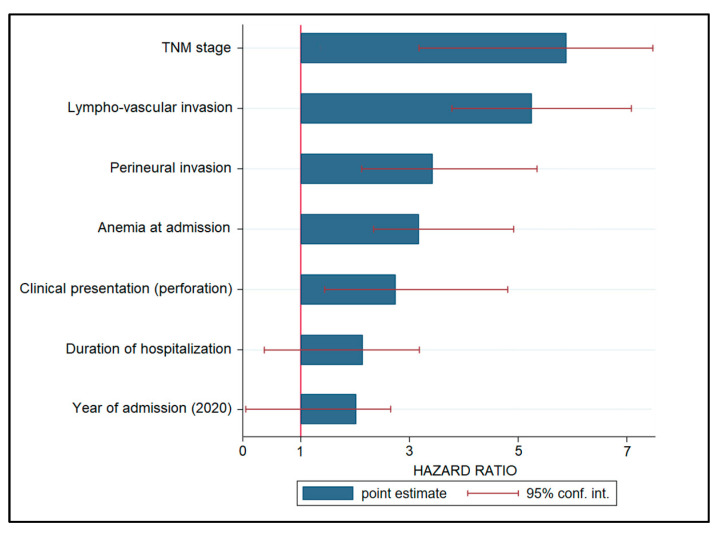
Risk factor analysis for disease progression in patients with colorectal cancer.

A Cox regression model was used to examine risk variables for cancer progression, and the hazard ratios (HR) are listed in decreasing order in Table 4 and Figure 3. Patients with an advanced TNM stage had a 6.28-fold increased risk of disease progression (*p* < 0.001), followed by lymphovascular invasion (HR = 5.19, *p* < 0.001). Other significant variables associated with disease development were perineural invasion (HR = 3.36), anemia during admission (HR = 3.20), and clinical presentation with bowel perforation (HR = 2.77). Other risk variables that were not statistically significant were hospitalization length (HR = 2.18) and intervention during the COVID-19 pandemic, represented by admission years 2020 and 2021 (HR = 1.94, CI = 0.17–2.79).

## 4. Discussion

### 4.1. Important Findings and Literature Review

This research examined how the Romanian SARS-CoV-2 pandemic affected colorectal cancer diagnosis and therapy. A considerable proportion of the oncological patients had a diminished medical attention throughout the ongoing SARS-CoV-2 pandemic, consistent with the estimations and forecasts. In addition, a significant number of patients who were initially diagnosed with colorectal cancer that was curable in its early stages may have become incurable in its later stages due to missed appointments for colorectal cancer screening and elective surgery, deliberate refusal of treatment, or deliberate delay of treatment out of fear of COVID-19 [25]. Although we observed a significantly higher proportion of stage IIa cancers in 2020, compared to the previous year and 2021, the difference is likely to happen just due to chance alone, since the overall comparison of colorectal cancer staging during the three-year study period was not statistically significant.

Our results are consistent with prior research, such as an investigation performed in the United States describing that, at the onset of the SARS-CoV-2 pandemic, it was advised that all non-urgent surgical and medical treatments, including screening colonoscopies, be postponed until the pandemic conditions stabilized [26]. This was also done to limit the danger of exposure to SARS-CoV-2, which is found in the feces of COVID-19 patients. In response, colorectal cancer screenings decreased by 90%, resulting in a 32% fall in new colorectal cancer diagnoses and a 53% decline in colorectal cancer-related surgical operations by mid-April 2020 [27]. In addition, by April 2021, the rate of regular screening colonoscopies remained 50 percent lower than pre-pandemic levels.

We observed a significant decrease in elective surgeries during 2020 and 2021, as compared with the year previous to the pandemic onset. The numbers dropped from 68.7% electives before the pandemic to 50.0% in 2020 and to the lowest value of 42.9% in 2021. This can be attributed to the increase in late presentations complicated by bowel obstruction, perforation, or rectal bleeding, which were significantly more common during the pandemic. However, this did not significantly influence in-hospital fatality, as described by other studies performed in Romania [28,29,30]. One study performed in the nation’s largest tertiary center for general surgery and emergency hospital compared the pre-pandemic and pandemic periods, observing a higher percentage of complex colorectal patients treated in an emergency (37% before the COVID-19 pandemic and 72% during the first year of the COVID-19 pandemic). They also discovered a considerably increased mortality in the pandemic group, which might be attributed to the larger proportion of acute complex cases since the emergency presentation of colorectal malignancies has a devastating effect on patient survival and should be avoided.

Compared to other studies that demonstrated a significant decrease in the management of cancer patients during the COVID-19 pandemic, as well as a decrease in healthcare accessibility, our center was able to maintain the same level of effort in managing colorectal cancer patients, even though the total number of interventions performed in our clinic decreased by 40.9% from 2.895 in 2019 to 1.709 in 2020. Only two patients with SARS-CoV-2 infection needed prompt surgical care while infected. These circumstances occurred because national recommendations issued during the COVID-19 pandemic made it clear that non-emergent surgical circumstances should be postponed until a negative PCR test from the patient is received. Other studies, however, reported that both the overall number of surgical interventions and the colorectal cancer surgeries decreased significantly, at least in the initial phase of the pandemic [31]

One meta-analysis that included more than 300,000 participants indicated that three of the seven studies found that a delay in elective resection is related to lower overall survival or disease-free survival rates [32]. This meta-analysis observed that when the overall survival was evaluated after a delay of one month, there was a 1.13 times higher likelihood of death, and when the survival was evaluated after a three-month delay, the combined risk was 1.57 times higher than the reference value. It was projected that the numbers needed to indicate harm would be 35 for a delay of one month and 10 for a delay of three months. According to the findings of the meta-analysis, a delay had a non-significantly unfavorable association with disease-free survival; therefore, individuals with colorectal cancer should not postpone elective surgery for more than four weeks since the information that is currently available shows that lengthy delays from the time of diagnosis are linked with inferior results [33]. It is essential to do targeted research in order to prioritize patient groups based on risk factors in the event of future delays or pandemics.

Regarding the risk factors for disease progression identified in this study, it was observed that the date of admission and surgical intervention during the first year of the pandemic did not represent a significant risk factor for disease progression and mortality, having an insignificant risk of 1.94. However, other risk factors, such as the TNM stage, lymphovascular invasion, perineural invasion, anemia at admission, and colic perforation at admission were all statistically significant risk factors for disease progression, with the highest likelihood being represented by an advanced TNM stage at admission, with a 6.28 times higher risk. One meta-analysis was able to confirm and quantify the considerable unfavorable effect that perineural invasion has on the prognosis of colorectal cancer patients in terms of disease-free survival and mortality [34]. The pooled hazard ratio for mortality from all the analyzed studies was 1.85. The DFS seemed to be the most impacted since the hazard ratio; in this case it was 2.35 times higher, which is comparable with the risk of 3.36 identified in our study. In conclusion, perineural invasion is a pathologic hallmark in colorectal cancer that has a significant influence on the patient’s prognosis, being comparable to other well-established prognostic variables such as the occurrence of lymph node metastases, depth of invasion, lymphatic invasion, vascular invasion, and differentiation grade.

The duration of hospitalization was also included in the risk factor analysis, although the risk did not show statistical significance. Moreover, it was observed that hospitalization was significantly lower during the two pandemic years, with the lowest being in 2021, decreasing from an average of 12.7 days per patient to 10.2 days. This is in accordance with a recent review describing the implications of the SARS-CoV-2 pandemic for elective surgery in patients suffering from colorectal cancer [35]. According to the findings of the study, both the amount of time spent in postoperative critical care and the total amount of time spent in the hospital should be reduced in order to lessen the risk of patient exposure and to make room for other patients to have surgery.

In order to reduce their risk of contracting the virus even further, patients should try to confine themselves to their homes for a period of at least 14 days prior to being admitted to the hospital. Surgeons preferred to shorten the hospitalization of patients for reasons such as reducing the risk of exposure to the new coronavirus, as well as due to patients’ fear of prolonged contact with the medical system. In general, the average length of hospitalization also decreased, with multiple studies showing a significant difference between the pandemic period and the previous period. This was due to the fact that the pandemic period was significantly different from the previous period [36].

### 4.2. Study Limitations and Future Perspectives

This study investigates the epidemiological characteristics, clinical features, and surgical characteristics of colorectal cancer patients in Romania. As a retrospective cohort design, the quality of the analyzed data might be lower since digitally created data from paper medical records is prone to human error. The second limitation is the small sample size included in the study. Consequently, these results may not completely and accurately reflect the characteristics and outcomes of individuals diagnosed with malignant cancer in Romania during the COVID-19 pandemic. Another limitation is the relatively short follow-up period that did not allow for a proper evaluation of the pandemic’s effects on disease-free survival and overall survival, although it was possible to compare only the disease progression at six months. As the probability of COVID-19 infection among healthcare workers was high, the danger of SARS-CoV-2 infection and the rise in COVID-19 patients may have impacted the registry capacity or data quality.

The present analysis provides conclusive evidence that the COVID-19 pandemic in Romania was not a major risk factor for disease progression among colorectal cancer patients. Therefore, the primary contribution is the consolidation of current information, and we advocate for the establishment of a threshold for an acceptable period of treatment deferral that should not compromise future outcomes and survival in colorectal cancer patients in surgical departments reorganized to support COVID-19 patients. Consequently, multicentric and large sample size investigations are required to identify the full spectrum of consequences caused by the continuing pandemic.

## 5. Conclusions

The existing literature suggests that the SARS-CoV-2 pandemic is responsible for a decrease in healthcare capability of cancer diagnosis and an increase in the number of treatment delays due to constraints of protecting the existing patients from SARS-CoV-2 infection, without increasing the proportions of advanced stages. However, the results from our clinic demonstrate that surgery for colorectal cancer can be performed without significant risks during the COVID-19 pandemic, but only if the SARS-CoV-2 infection prevention guidelines are strictly adhered to. Even while the overall number of elective surgical operations reduced throughout the pandemic years, the number of interventions for colorectal cancer remained stable. However, our single-center research does not exclude the issue that there may have been a significant increase in the number of colorectal cancer cases that were not correctly diagnosed, which may be associated with poorer outcomes and greater mortality rates in the coming years. Therefore, healthcare systems can prepare for the future by implementing special departments for managing contagious patients instead of reorganizing surgical-oncological departments.

## Figures and Tables

**Table 1:** Baseline characteristics of patients with colorectal cancer stratified by the year of initial surgical intervention.

	2019 (*n* = 83)	2020 (*n* = 80)	2021 (*n* = 84)	*p*-Value *
Background				
Age, years (mean ± SD)	67.2 ± 10.3	64.6 ± 10.9	66.8 ± 10.6	0.250
Age range				0.874
<50	2 (2.4%)	9 (11.3%)	6 (7.1%)	
50–70	54 (65.1%)	46 (57.5%)	53 (63.1%)	
≥71	27 (32.5%)	25 (31.3%)	25 (29.8%)	
Sex				0.189
Female	31 (37.3%)	29 (36.3%)	41 (48.8%)	
Male	52 (62.7%)	51 (63.8%)	43 (51.2%)	
BMI, kg/m^2^ (mean ± SD)	26.3 ± 3.9	27.5 ± 4.1	26.9 ± 3.7	0.147
Substance use behavior				
Chronic smoking	25 (30.1%)	27 (33.8%)	29 (34.5%)	0.812
Chronic alcohol use	6 (7.2%)	5 (6.3%)	8 (9.5%)	0.720
Place of origin				0.626
Rural	39 (47.0%)	42 (52.5%)	38 (45.2%)	
Urban	44 (53.0%)	38 (47.5%)	46 (54.8%)	
Occupation				0.099
Employed	22 (36.5%)	34 (42.5%)	24 (28.6%)	
Unemployment	7 (8.4%)	10 (12.5%)	8 (9.5%)	
Retired	54 (65.1%)	36 (45.0%)	52 (61.9%)	
Civil status				0.455
Married	76 (91.6%)	72 (90.0%)	72 (85.7%)	
Single/Divorced/Widowed	7 (8.4%)	8 (10.0%)	12 (14.3%)	
Referred from				0.114
Primary care	39 (47.0%)	25 (31.3%)	35 (41.7%)	
Secondary care	44 (53.0%)	55 (68.8%)	49 (58.3%)	
SARS-CoV-2 infection	-	2 (2.5%)	2 (2.4%)	0.960

* Chi-square or Fisher’s exact test; SD—standard deviation.

**Table 2:** Comparison of colorectal cancer characteristics during the study period.

	2019 (*n* = 83)	2020 (*n* = 80)	2021 (*n* = 84)	*p*-Value
Number of comorbidities				0.370
0–1	27 (32.5%)	30 (37.5%)	24 (28.6%)	
2	42 (50.6%)	44 (55.0%)	48 (57.1%)	
≥3	14 (16.9%)	6 (7.5%)	12 (14.3%)	
Anatomical distribution				0.506
Colic	50 (60.2%)	48 (60.0%)	44 (52.4%)	
Rectal	33 (39.8%)	32 (40.0%)	40 (47.6%)	
Chemo/Radiotherapy before admission	6 (7.2%)	5 (6.3%)	8 (9.5%)	0.720
Grading				0.247
I	5 (6.0%)	1 (1.3%)	2 (2.4%)	0.195
II	7 (8.4%)	2 (2.5%)	4 (4.8%)	0.229
III	59 (71.1%)	66 (82.5%)	63 (75.0%)	0.222
IV	12 (14.5%)	10 (12.5%)	16 (19.0%)	0.488
TNM staging				0.120
0	5 (6.0%)	1 (1.3%)	3 (3.6%)	0.266
I	12 (14.5%)	15 (18.8%)	10 (11.9%)	0.464
IIa	11 (13.3%)	23 (28.8%)	11 (13.1%)	0.012
IIb	3 (3.6%)	2 (2.5%)	3 (3.6%)	0.901
IIc	2 (2.4%)	1 (1.3%)	3 (3.6%)	0.627
IIIa	1 (1.2%)	2 (2.5%)	0 (0.0%)	0.343
IIIb	28 (33.7%)	16 (20.0%)	24 (28.6%)	0.140
IIIc	11 (13.3%)	10 (12.5%)	9 (10.7%)	0.875
IV	10 (12.0%)	10 (12.5%)	21 (25.0%)	0.038
Aggressiveness				
Lymphovascular invasion	42 (50.6%)	41 (51.3%)	51 (60.7%)	0.341
Perineural invasion	55 (33.7%)	53 (33.8%)	48 (42.9%)	0.371

Data analyzed using chi-square or Fisher’s exact test; TNM—tumor-node-metastasis.

**Table 3:** Comparison of colorectal cancer surgery during the study period.

	2019 (*n* = 83)	2020 (*n* = 80)	2021 (*n* = 84)	*p*-Value *
Proportion of CRC surgical interventions in the clinic	83 (2.86%)	80 (4.68%)	84 (4.49%)	0.001
Elective surgery	57 (68.7%)	40 (50.0%)	36 (42.9%)	0.002
Emergency presentation	(*n* = 26)	(*n* = 40)	(*n* = 36)	0.012
Bowel obstruction	17 (65.4%)	32 (80.0%)	37 (75.0%)	
Perforated bowel	0 (0.0%)	4 (10.0%)	6 (16.7%)	
Rectal bleeding	9 (34.6%)	4 (10.0%)	5 (8.3%)	
Type of surgery				0.178
Open resection	57 (68.7%)	61 (76.3%)	68 (81.0%)	
Laparoscopy	26 (31.3%)	19 (23.8%)	16 (19.0%)	
Days of hospitalization	12.7 ± 8.5	10.6 ± 3.3	10.2 ± 7.5	0.038
Outcomes				
Colostomy	13 (15.7%)	22 (27.5%)	23 (27.4%)	0.119
In-hospital mortality	4 (4.8%)	6 (7.5%)	4 (4.8%)	0.689
Disease progression at six months	19 (22.9%)	25 (31.3%)	26 (31.0%)	

* Chi-square or Fisher’s exact test; DFS—disease-free survival; CRC—colorectal cancer.

**Table 4:** Risk factors for colorectal cancer progression after the initial hospital visit.

Risk Factors	HR	CI	*p*-Value
Advanced TNM stage	6.28	3.17–7.30	<0.001
Lymphovascular invasion	5.19	3.46–7.08	<0.001
Perineural invasion	3.36	1.72–4.81	<0.001
Anemia at admission	3.20	2.14–4.96	<0.001
Clinical presentation (perforation)	2.77	1.25–4.90	0.001
Duration of hospitalization	2.18	0.43–3.26	0.033
Year of admission (2020)	1.94	0.17–2.79	0.042

TNM—tumor-node-metastasis; HR—hazard ratio; CI—confidence interval.

## Data Availability

The data presented in this study are available on request from the corresponding author.
